# Distinctive Traits for Drought and Salt Stress Tolerance in Melon (*Cucumis melo* L.)

**DOI:** 10.3389/fpls.2021.777060

**Published:** 2021-11-04

**Authors:** Sergio Chevilly, Laura Dolz-Edo, Gema Martínez-Sánchez, Luna Morcillo, Alberto Vilagrosa, José M. López-Nicolás, José Blanca, Lynne Yenush, José M. Mulet

**Affiliations:** ^1^Instituto de Biología Molecular y Celular de Plantas, Universitat Politècnica de València-Consejo Superior de Investigaciones Científicas, Valencia, Spain; ^2^Fundación Centro de Estudios Ambientales del Mediterráneo, Joint Research Unit University of Alicante–Centro de Estudios Ambientales del Mediterráneo (CEAM), University of Alicante, Alicante, Spain; ^3^Departamento de Bioquímica y Biología Molecular-A, Facultad de Biología, Universidad de Murcia, Murcia, Spain; ^4^Instituto Universitario de Conservación y Mejora de la Agrodiversidad Valenciana, COMAV, Universitat Politècnica de València, Valencia, Spain

**Keywords:** melon, *Cucumis melo*, salt stress, drought stress, amino acids, plant hormones, ion content

## Abstract

Melon (*Cucumis melo* L.) is a crop with important agronomic interest worldwide. Because of the increase of drought and salinity in many cultivation areas as a result of anthropogenic global warming, the obtention of varieties tolerant to these conditions is a major objective for agronomical improvement. The identification of the limiting factors for stress tolerance could help to define the objectives and the traits which could be improved by classical breeding or other techniques. With this objective, we have characterized, at the physiological and biochemical levels, two different cultivars (sensitive or tolerant) of two different melon varieties (Galia and Piel de Sapo) under controlled drought or salt stress. We have performed physiological measurements, a complete amino acid profile and we have determined the sodium, potassium and hormone concentrations. This has allowed us to determine that the distinctive general trait for salt tolerance in melon are the levels of phenylalanine, histidine, proline and the Na^+^/K^+^ ratio, while the distinctive traits for drought tolerance are the hydric potential, isoleucine, glycine, phenylalanine, tryptophan, serine, and asparagine. These could be useful markers for breeding strategies or to predict which varieties are likely perform better under drought or salt stress. Our study has also allowed us to identify which metabolites and physiological traits are differentially regulated upon salt and drought stress between different varieties.

## Introduction

Melon (*Cucumis melo L*.) is a major crop with great agronomic and economic interest, considered a gourmet food in several markets and cultures. One of the main problems for melon farming is that its cultivation demands a lot of water (Cabello et al., 2009). In the current context of anthropogenic global warming and the subsequent climate change, aridity is increasing in traditional cultivation areas, and thus, melon culture is subjected to increasing abiotic stress, which compromises the yield. Specifically, drought stress is increasing, and salt stress is directly related to this water scarcity, given that excessive irrigation increases the salt deposition in the soil and diminishes the phreatic level, thus enabling the infiltration of sea water. It is estimated that 20% of all arable land and almost half of the land with water availability are affected by salts, significantly reducing yield below the genetic potential of most crops (Botella et al., 2007; Chandna et al., 2014). As a result of salinization, crop yields are declining while arable land is being irreversibly lost (Nawaz et al., 2010). High salinity levels also increase soil pH. In addition, saline stress leads to deterioration of soil structure and prevents the air-water balance, essential for biological processes occurring in the roots (Galvan-Ampudia et al., 2013). Saline soils reduce the biomass production of crops affecting important biochemical and physiological processes in the plant (Serrano et al., 1999).

We have generated considerable knowledge at the biochemical level and physiological level regarding how abiotic stress affects basic physiological processes, the cellular function and even the biochemical targets, but there are still large gaps in our knowledge about the limiting factors for stress responses. More specifically, we are lacking knowledge regarding which traits could be improved by breeding or genetic engineering that would have a major impact on plant growth and development under stress conditions. This explains the low success in breeding novel crops that are adapted to saline soils or are able to maintain yield under drought stress conditions (Ashraf et al., 2009). Proof of this scarcity of results is that there are only two GMO cultivars on the market whose trait is drought tolerance: the Droughtgard maize from BASF and the HB4 soy from Agroceres (Wang et al., 2015; Ribichich et al., 2020). To date, there are no marketed biotechnological crops with enhanced yield under saline conditions.

Several strategies have been developed to identify the limiting factors for stress tolerance. Evaluating the physiological and biochemical response of stress tolerant and stress sensitive plants is a well-established strategy to discover differential traits for abiotic stress tolerance (Taibi et al., 2017, 2018; Chevilly et al., 2021). All these analyses have been performed testing different cultivars from the same variety and a single stress. We have further developed this concept by evaluating, in the same analysis, different stresses and different cultivars of two different varieties to find limiting factors which are not particular to a specific variety or stress. In this report, we have applied this strategy to a pivotal horticultural crop for the economy in the Mediterranean area. There are several reports evaluating Galia melon performance under salt stress in field conditions (Akrami and Arzani, 2018; Akrami et al., 2019), but so far, there are no studies evaluating the plant response at the initial stages of development under controlled conditions. This work has been designed to determine the differences at the physiological and biochemical levels between different melon genotypes under two different abiotic stresses. These varieties had previously been characterized as sensitive or tolerant to abiotic stress. We have subjected these varieties to controlled drought or salinity stress, and have monitored different physiological or biochemical parameters, in order to find changes that are relevant among varieties or treatments. This will allow us to identify the limiting factors in abiotic stress tolerance and will help to define novel breeding strategies.

## Materials and Methods

### Plant Material

The four varieties of pre-commercial melon *(Cucumis melo* L.) seeds used were provided by Enza Zaden and referred to as Cv. 1, Cv. 2, Cv. 3, and Cv. 4. Cv. 1 is a Galia melon (*Cucumis melo* Cv. *reticulatus*) tolerant to abiotic stress, Cv. 2 is a Galia type melon sensitive to abiotic stress; Cv. 3 melon is a Piel de Sapo (a.k.a. Santa Claus Melon; *Cucumis melo* Cv. *inodorus*) tolerant to abiotic stress; and Cv. 4 is a Piel de Sapo sensitive to abiotic stress.

### Experimental Design

For different experiments, 20 seeds of each variety were germinated in a Petri dish with moist sterile Whatmann filter paper. After 5 days, seedlings were transferred to a substrate (50% kekkila peat, 25% perlite, 25% vermiculite) in individual plant pots 12 cm diameter × 8 cm height. The experimental design consisted of an aleatory placement where each block was composed by 4 pots per tray and one plant per pot. Each experiment consisted in 5 individuals × 4 varieties × 3 treatments (60 total plants). Plants were watered with Hoagland solution. After 3 weeks, when plants reached the four-leaf phase, irrigation was maintained (control plants), limited (drought stress) or watered with Hoagland solution plus 220 mM NaCl (salt stress). Samples were taken or measurements were performed after 6 days of stress treatment (salt stress) or when the total weight (plant and container) was reduced to 60% of their initial weight (drought stress), at about 9 days. In all cases, the number of samples per experiment (n) refers to biological replicates from different plants (between 3 and 5). All samples for each treatment were collected at the same time. Plants were grown in a phytotron at 25 ± 2°C, humidity of 50–60% and a photoperiod of 16 h light/8 h darkness (200 μmol m^−2^ s^−1^ of light intensity). All experiments were replicated to check the reproducibility of the results.

### Physiological Measurements

The water potential (Ψ_w_, MPa) was measured with a Schölander pressure pump (model PMS-1000, PMS Instruments, Corvallis, OR, United States). Stomatal conductance (*g*_s_, mmol H_2_O m^−2^s^−1^), the sub-stomatal concentration of CO_2_ (C_i_), photosynthetic rate (A, μmol CO_2_ m^−2^s^−1^), transpiration (E, mmol H_2_O m^−2^s^−1^), water use efficiency (WUE, μmol CO_2_ mmol^−1^H_2_O) and leaf temperature through infrared Thermometry (Tleaf, °C), were determined with a CIRAS-3 portable photosynthesis system (PP Systems, Amesbury MA). The measurements were recorded under saturating light conditions (1,500 μmol quanta m^−2^ s^−1^), with a temperature of 25°C, and ambient CO_2_ concentration of 400 mol^−1^ CO_2_ and a relative humidity of ~55%. Chlorophyll fluorescence indices (i.e., Fv/Fm and Quantum yield) were measured with a portable pulse-amplitude modulated chlorophyll fluorometer (PAM-2100, Heinz Walz, Effeltrich, Germany). These measurements of the photosystem II efficiency were performed once the plants were adapted to darkness for 30 min, on the same leaves where stomatal conductance and photosynthesis were determined. All measures were performed on the third youngest full-developed leaf of each plant, analyzing a total of five plants per variety.

### Amino Acid Analysis

One gram of the third youngest leaf was taken, lyophilized and ground with a mortar and pestle in the presence of liquid nitrogen. The resulting powder was homogenized for 30 s with 2 mL of 2% citrate buffer pH 2 (Mulet et al., 2004) and centrifuged for 5 min at 13,000 *g*. The supernatant was filtered through a 25-micrometer pore-size non-sterile filter. 1/10 dilutions of these extracts were injected into an automatic Beckman Gold amino acid analyzer. The analysis was carried out according to the protocol supplied by the manufacturer, using a system of ninhydrin and sodium citrate for detection. Measurements were normalized to dry weight.

### Hormone Quantification

Plant hormones were determined following the method of Durgbanshi (Durgbanshi et al., 2005). Briefly, lyophilized samples were ground to powder in the presence of liquid nitrogen. Two hundred milligram per replicate were purified with solid phase extraction columns (SPE; reverse phase and ion exchange), using internal deuterated standards. The analysis was carried out using UPLC-mass spectrometry (Acquity SDS, Waters Corp., Milford, MA). Measurements were normalized to dry weight.

### Ion Content Determination

Ions were determined as described (Gisbert et al., 2020). Briefly, samples of the third youngest leaf from 1-month-old plants (about 1 g) were dried at 70°C for 4 days. Dry weight was determined, and ions were extracted by a 30 min incubation in 1 mL of 0.1M HNO_3_ at room temperature. Then samples were centrifuged, and the supernatant was diluted with 4 mL of milliQ water and filtered (22 μM). Sodium and potassium were measured in a plasma emission spectrophotometer (Shimadzu), as described (Rios et al., 2012). Measurements were normalized to dry weight.

### Statistical Analysis

The ANOVA was performed by using the SPSS software v.25.0 statistical package (IBM SPSS Statistics for Windows, Armonk, NY, USA; IBM Corp.). The means were considered to be significantly different at *p* < 0.05 after Duncan's new multiple range test (MRT) (Duncan, 1955).

## Results

### Physiological Determinations

**Figure 1:**
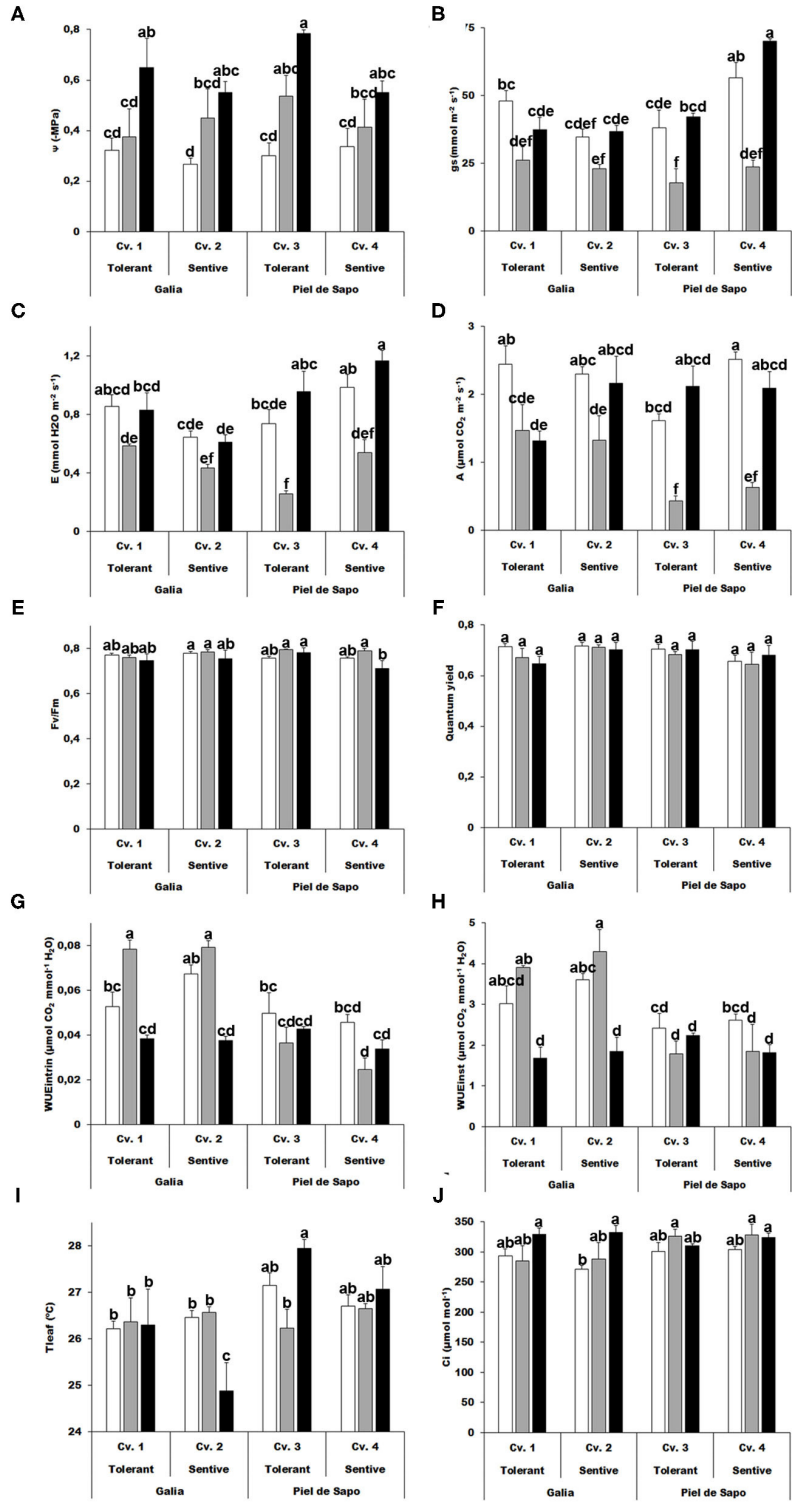
Physiological measurements. Water potential (Ψw) **(A)**; stomatal conductance (gs) **(B)**; transpiration (E) **(C)**; Net photosynthesis (A) **(D)**; Quantum efficiency of photosystem II (Fv/Fm) **(E)**; Quantum yield **(F)**; intrinsic water use efficiency (WUEintr) **(G)**; instantaneous water use efficiency (WUEinst) **(H)**; Leaf temperature (Tleaf) **(I)** and sub-stomatal CO_2_ concentration (Ci) **(J)** of Galia tolerant genotype (Cv. 1), Galia sensitive genotype (Cv. 2), Piel de Sapo tolerant genotype (Cv. 3) or Piel de Sapo sensitive genotype (Cv. 4) under control (white bars), salt stress (gray bars) and drought stress (black bars) conditions. Data with different letters differ significantly (*p* < 0.05), as determined by Duncan's MRT test (*n* = 5). Scale bars are the mean ± standard error (SE). Experiment was replicated with similar results.

Several responses of plants to abiotic stress occur at the physiological level. We investigated whether we could identify differential responses among varieties or cultivars. As expected, the water potential increased upon stress between 1.12 and 1.25 for salt stress and 1.3 and 2.67 for drought stress (expressed as –MPa; Figure 1A), thus validating our experimental design. The tolerant cultivars presented higher values. The salinity treatment had a negative effect on stomatal conductance (gs), while the drought stress had a more modest effect on this parameter, observing minor differences when compared to the corresponding control (Figure 1B). A similar pattern was found with transpiration (E) and photosynthesis (A), which was stable upon drought stress, but decreased upon salt stress. Interestingly A also decreased upon drought stress in the tolerant Galia cultivar (Figures 1C,D). Maximum efficiency of photosystem II (determined as Fv/Fm) and quantum yield presented minor, but in some cases significant changes (Figures 1E,F). Water Use efficiency, intrinsic and instantaneous, decreased under drought stress and increased upon salt stress in Galia plants, but was stable in Piel de Sapo (Cv. 3; Figures 1G,H).

We also determined the leaf temperature and found a differential response among varieties. In Galia, the leaf temperature decreased in the tolerant variety upon drought stress about 4%, and in the Piel de Sapo the leaf temperature increased in the tolerant variety about 0,3% (Figure 1I). We observed minor effects on the sub-stomatical CO_2_ concentration (Ci) (Figure 1J).

### Amino Acid Measurements

**Figure 2:**
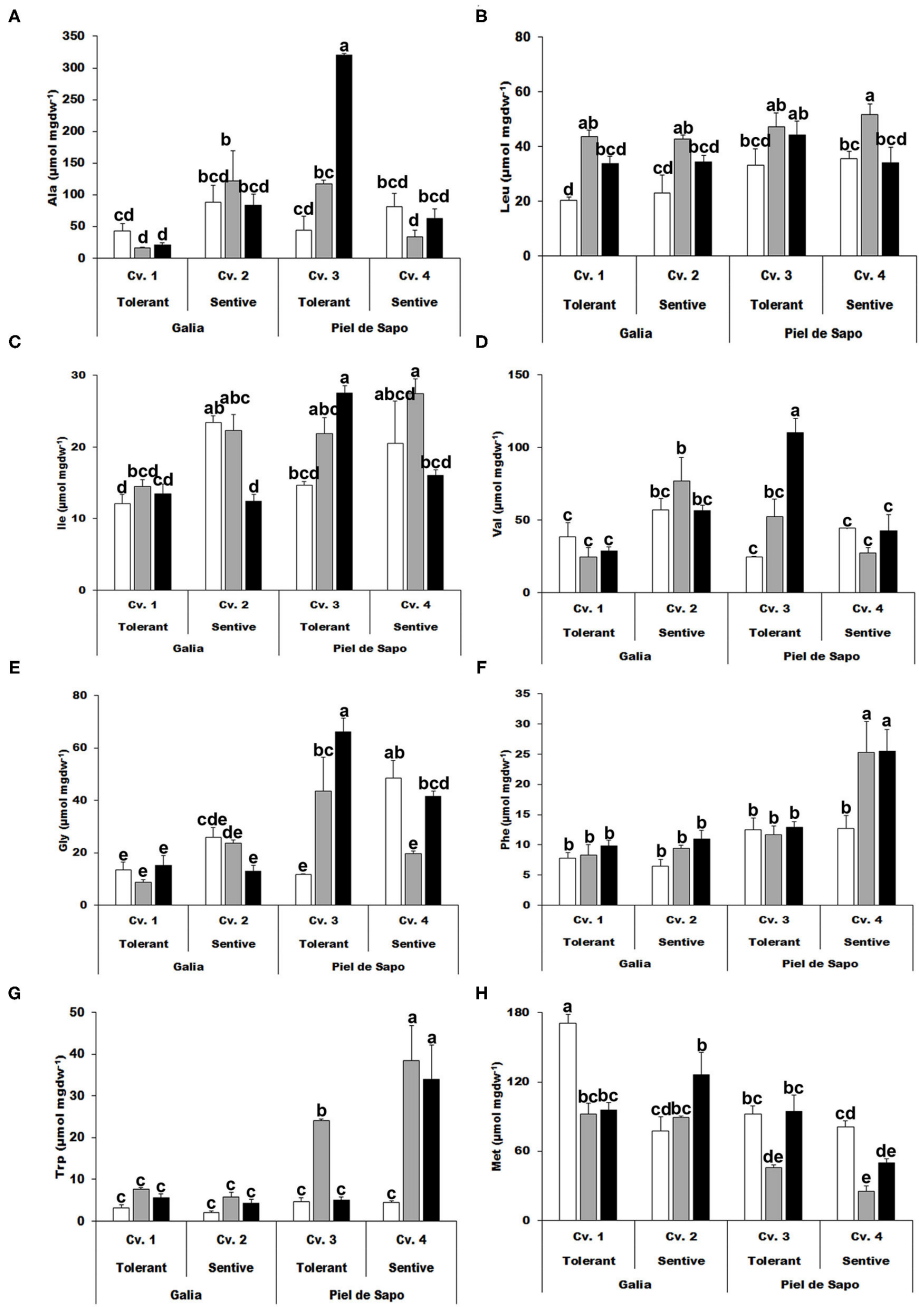
Hydrophobic amino acids. Alanine (Ala) **(A)**; leucine (Leu) **(B)**; isoleucine (Ile) **(C)**; valine (Val) **(D)**; glycine (Gly) **(E)**; phenylalanine (Phe) **(F)**; tryptophan (Trp) **(G)**; and methionine (Met) **(H)** of Galia tolerant genotype (Cv. 1), Galia sensitive genotype (Cv. 2), Piel de Sapo tolerant genotype (Cv. 3) or Piel de Sapo sensitive genotype (Cv. 4) under control (white bars), salt stress (gray bars) and drought stress (black bars) conditions. Data with different letters differ significantly (*p* < 0.05), as determined by Duncan's MRT test (*n* = 3). Scale bars are mean ± SE. Experiment was replicated with similar results.

Once we had studied the response of the selected varieties and cultivars at the physiological level, we further investigated the level of amino acids. First, we focused on the hydrophobic amino acids (Figure 2). In most cases, there was no distinctive pattern. However, for leucine (Leu), the concentration increased under salt stress (between 40 and 115%), and to a minor extent, under drought stress (between 0 and 67%) (Figure 2B). Glycine (Gly) can act as an osmolyte and is a precursor of antioxidant molecules, such as the tripeptide glutathione. Its concentration under stress conditions correlated with tolerance to stress, but only for the Piel the Sapo variety (Figure 2E). Similarly, phenylalanine (Phe) concentrations under drought stress correlated with sensitivity (Figure 2F). Finally, we observed a 7 fold increase in Tryptophan (Trp) concentration in Piel de Sapo sensitive cultivar under drought stress conditions (Figure 2G).

**Figure 3:**
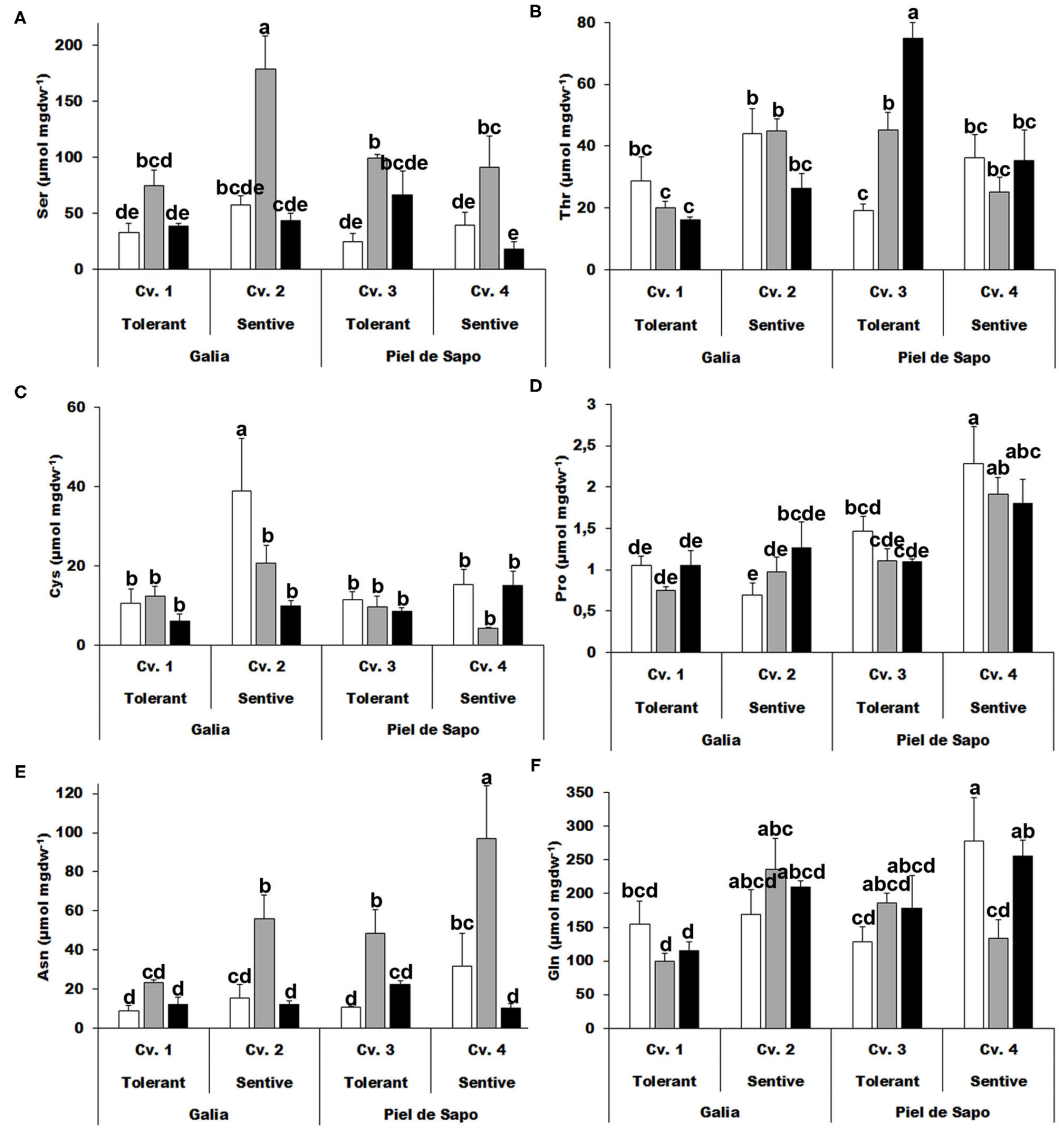
Polar amino acids. Serine (Ser) **(A)**; threonine (Thr) **(B)**; cysteine (Cys) **(C)**; proline (Pro) **(D)**; asparagine (Asn) **(E)**; and glutamine (Gln) **(F)** of Galia tolerant genotype (Cv. 1), Galia sensitive genotype (Cv. 2), Piel de Sapo tolerant genotype (Cv. 3), or Piel de Sapo sensitive genotype (Cv. 4) under control (white bars), salt stress (gray bars), and drought stress (black bars) conditions. Data with different letters differ significantly (*p* < 0.05), as determined by Duncan's MRT test (*n* = 3). Scale bars are mean ± SE. Experiment was replicated with similar results.

We further investigated the polar amino acids. Serine (Ser) concentrations increased, between 2.3- to 4-fold, under salt stress (Figure 3A) and similar results were obtained for asparagine (Asn). For other amino acids, such as threonine (Thr), cysteine (Cys), proline (Pro), or glutamine (Gln), we did not find a distinctive pattern (Figure 3).

**Figure 4:**
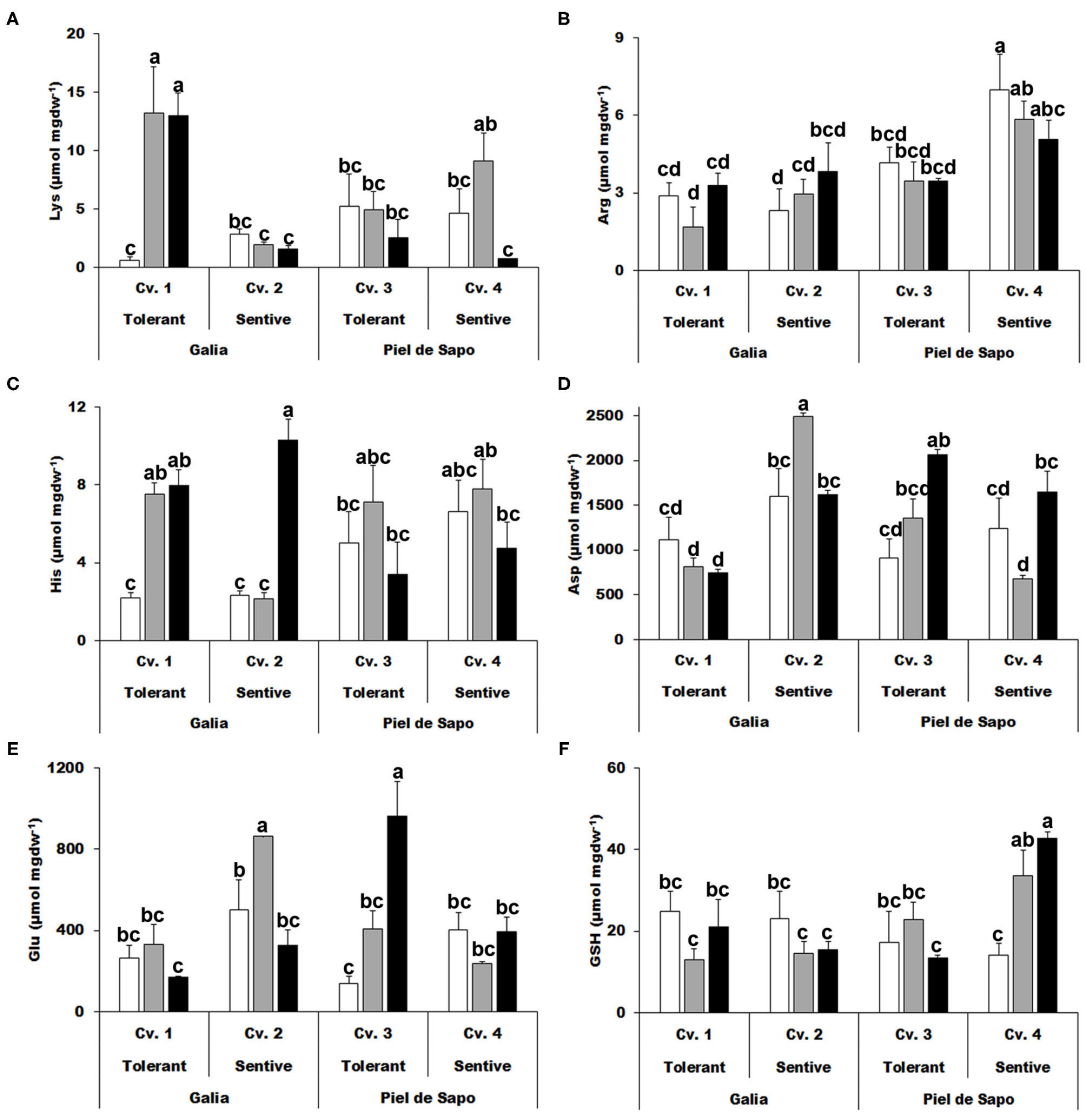
Charged amino acids. Lysine (Lys) **(A)**; arginine (Arg) **(B)**; histidine (His) **(C)**; aspartic acid (Asp) **(D)**; glutamic acid (Glu) **(E)**; and glutathione (GSH) **(F)** concentrations of Galia tolerant genotype (Cv. 1), Galia sensitive genotype (Cv. 2), Piel de Sapo tolerant genotype (Cv. 3), or Piel de Sapo sensitive genotype (Cv. 4) under control (white bars), salt stress (gray bars), and drought stress (black bars) conditions. Data with different letters differ significantly (*p* < 0.05), as determined by Duncan's MRT test (*n* = 3). Scale bars are mean ± SE. Experiment was replicated with similar results.

We also studied the charged amino acids and found that an increase in the levels of lysine (Lys) correlate with salt tolerance, but for drought tolerance only in the case of the Galia cultivar (Figure 4A). Also, an approximate 4-fold increase of histidine (His) concentration was observed under drought stress conditions for Galia cultivars (Figure 4C). Aspartic acid (Asp) levels behaved in disparate manners: in Galia they increased under salt stress in the sensitive cultivar (Cv. 2), while in Piel de Sapo, they increased in the tolerant cultivar (Cv. 3; Figure 4D). Glutamic acid (Glu) levels increased under salt and drought stress with respect to the control only in Piel de Sapo (Figure 4E). We did not find a distinctive pattern for GSH (Figure 4F).

### Sodium and Potassium Content

**Figure 5:**
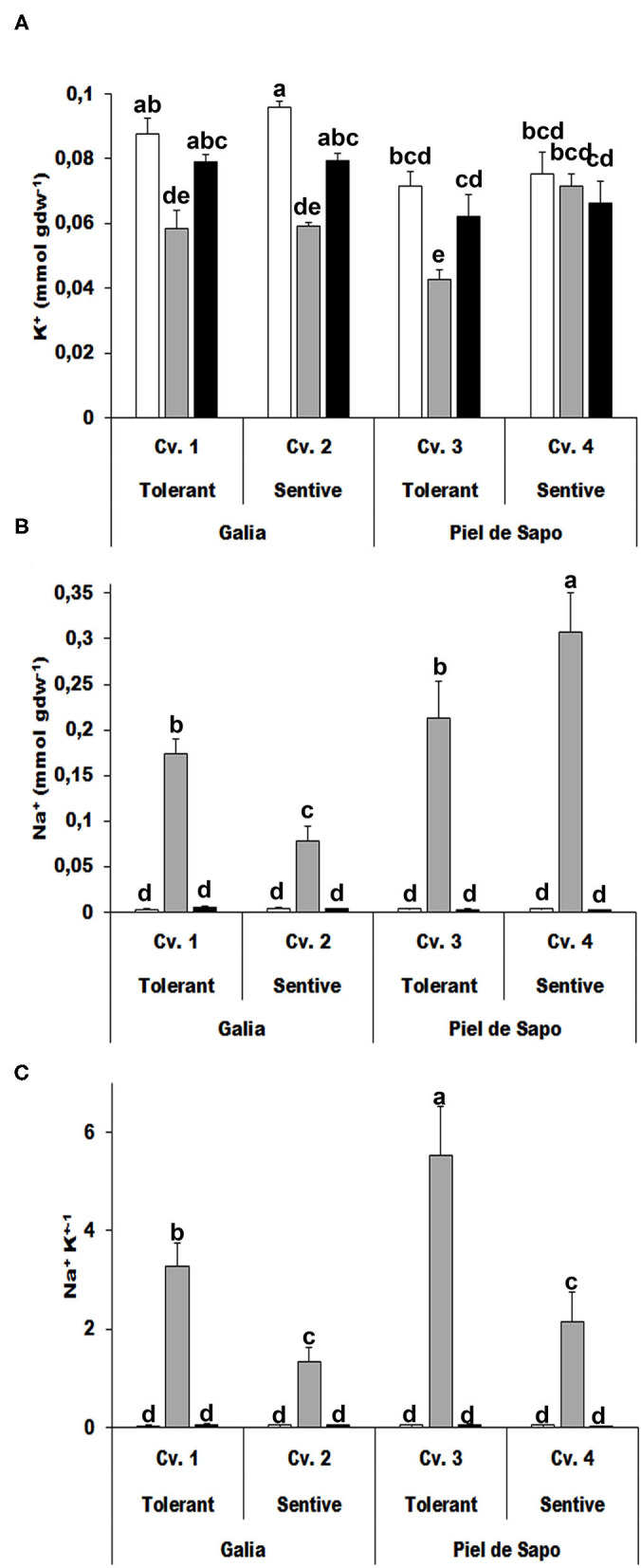
Ion content determination. Potassium content (K^+^) **(A)**, sodium content (Na^+^) **(B)**, and the Na^+^/K^+^ ratio **(C)** of the tolerant Galia genotype (Cv. 1), sensitive Galia genotype (Cv. 2), tolerant Piel de Sapo genotype (Cv. 3), or sensitive Piel de Sapo genotype (Cv. 4) under control (white bars), salt stress (gray bars), and drought stress (black bars) conditions. Data with different letter differ significantly (*p* < 0.05), as determined by Duncan's MRT test (*n* = 5). Scale bars are mean ± SE. Experiment was replicated with similar results.

We determined the ion content of the investigated varieties and cultivars under control and stress conditions. As expected, the potassium concentration decreased under salt stress conditions (between 5 and 40%), as sodium competes with potassium. Under drought stress, the potassium concentration also decreased about 10%. Potassium has been described to act as an osmolyte, but according to our results, that is not its main role in the investigated plants (Figure 5A). Sodium concentrations behaved differently depending on the variety. Sodium levels were higher in the tolerant cultivar in Galia plants, while the levels were lower in tolerant cultivars in Piel de Sapo plants (Figure 5B). In all cases, the Na^+^/K^+^ ratio was higher for tolerant cultivars (Figure 5C).

### Hormone Determination

**Figure 6:**
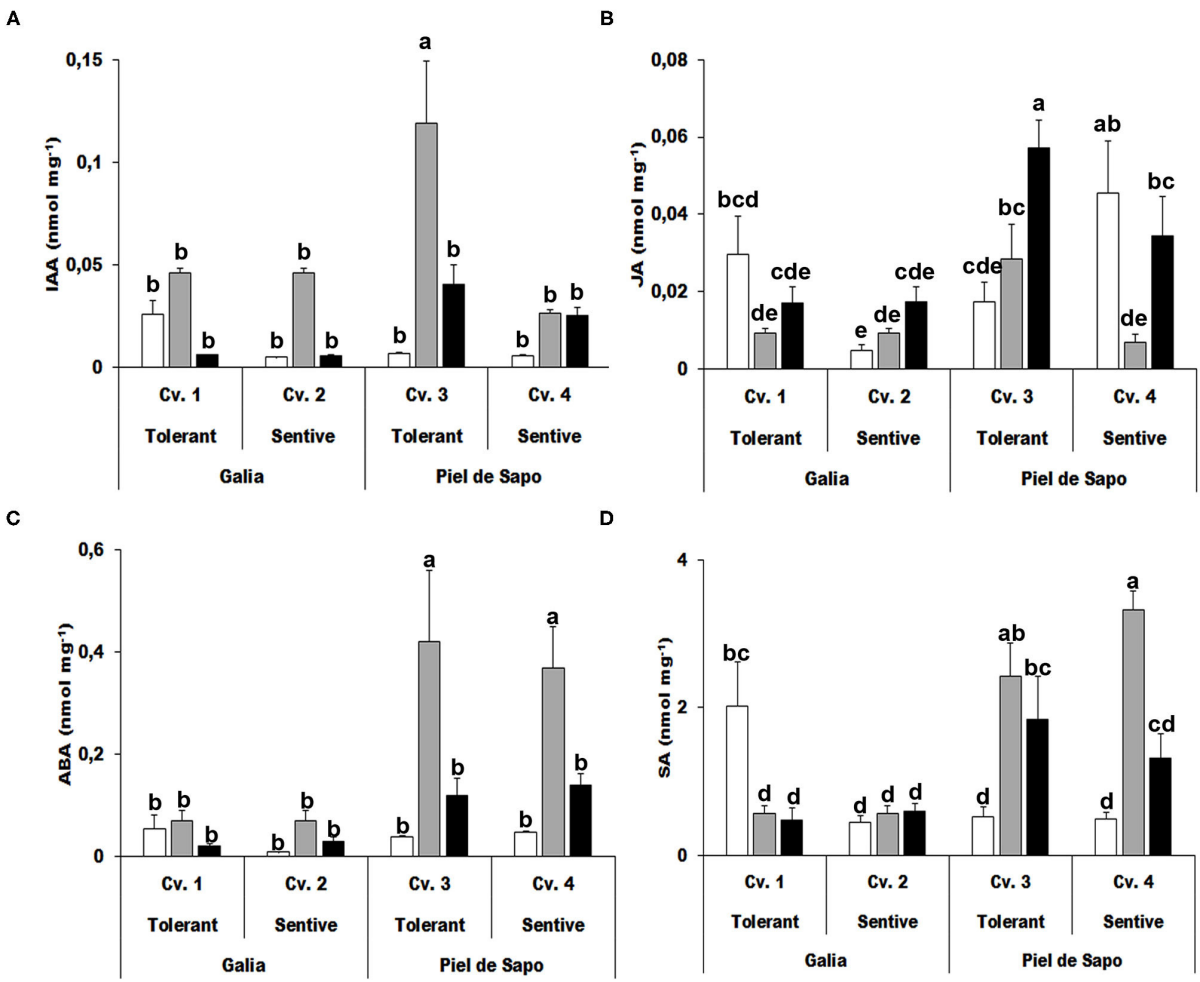
Hormone concentrations. Indolacetic acid (IAA) **(A)**; jasmonic acid (JA) **(B)**; abscisic acid (ABA) **(C)**, and salicylic acid (SA) **(D)** concentrations of Galia tolerant genotype (Cv. 1), Galia sensitive genotype (Cv. 2), Piel de Sapo tolerant genotype (Cv. 3), or Piel de Sapo sensitive genotype (Cv. 4) under control (white bars), salt stress (gray bars), and drought stress (black bars) conditions. Data with different letters differ significantly (*p* < 0.05), as determined by Duncan's MRT test (*n* = 5). Scale bars are mean ± SE. Experiment was replicated with similar results.

One of the most determinant aspects of stress tolerance is the hormonal response. Hormones, such as abscisic acid (ABA) or salicylic acid (SA), are directly involved in the response to abiotic stress, while other hormones, such as indolacetic acid (IAA) or jasmonic acid (JA) are mainly related to growth, but indirectly may affect the response to abiotic stress. We determined the levels of different hormones under control and stress conditions. IAA concentrations increased 17-fold under salt stress in the tolerant cultivar of the Piel de Sapo variety (Figure 6A). Levels of JA decreased upon stress in the tolerant Galia cultivar (70% for salt stress and 43% in drought stress) and increased (1.91-fold for salt stress and 3.53-fold drought stress) in the sensitive cultivar (Figure 6B). As expected, ABA levels increased upon stress, but the increase was more pronounced in Piel de Sapo plants under salt stress (between 8 and 11 fold) (Figure 6C). SA levels also increased upon stress but, again, only in Piel de Sapo plants (Figure 6D).

## Discussion

**Figure 7:**
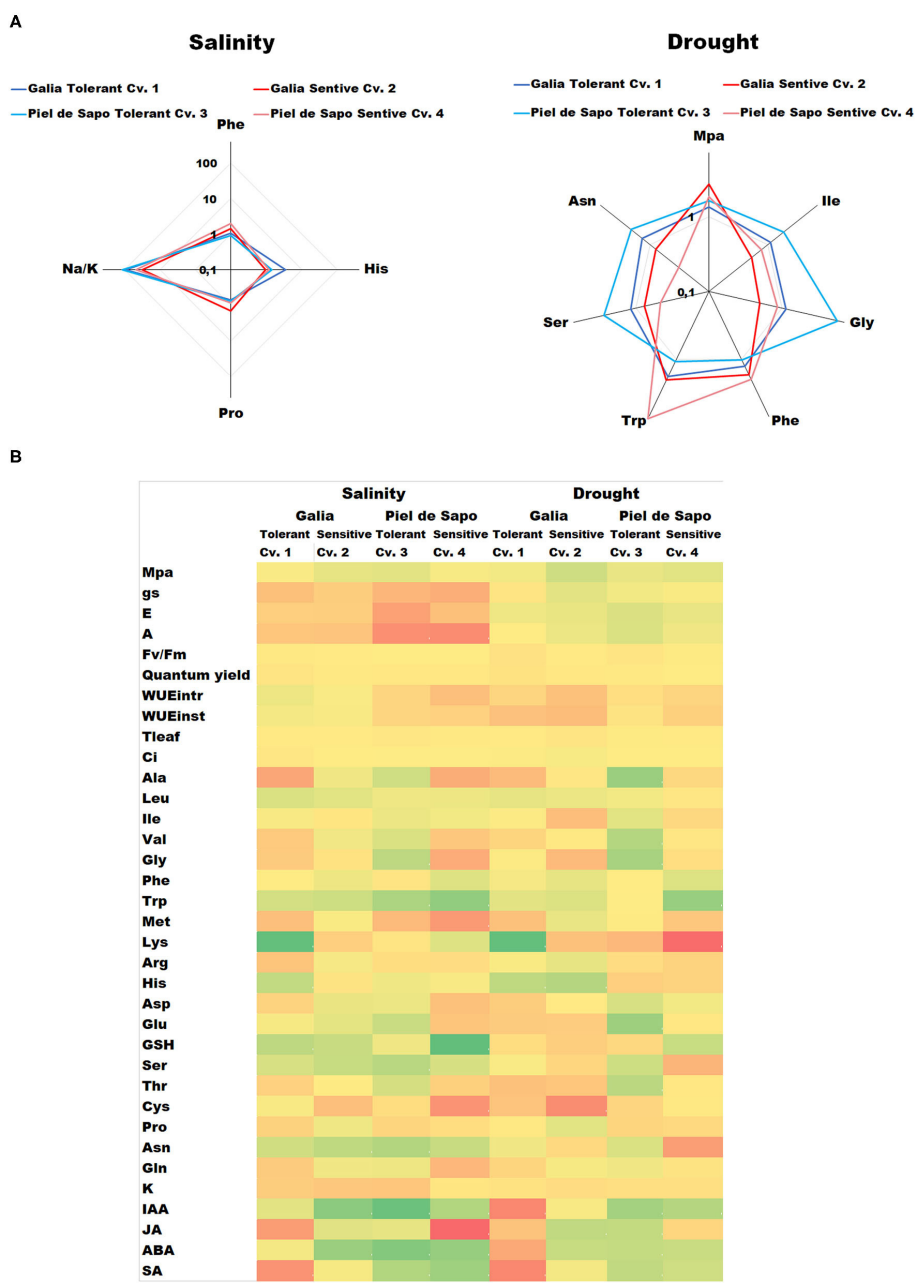
Summary of the main findings of this study. **(A)** Radial diagrams of the ratio between stress/control concentration under salt stress (left) or stress/control concentration under drought stress (right). The tolerant cultivars are shown in shades of blue and the sensitive cultivars in shades of red. The values are represented in a decimal logarithmic scale. **(B)** Heat map of all the results obtained in the present study. Green indicates higher stress/control values, yellow average stress/control values while red indicates lower stress/control values in a decimal logarithmic scale.

The main objective of this study is to compare physiological and biochemical responses of two cultivars of two different varieties, to both salt and drought stress, in order to find common patterns among different varieties. We included stress tolerant and sensitive cultivars as well to gain further insight into differential responses within varieties. Through the relativization of data (i.e., the ratio of the value under stress with respect to the value under control conditions) we have found that, irrespectively of the variety, tolerance to salt stress correlates with higher ratios (stress/control) for His (3.4 and 1.42 for tolerant vs. 0.92 and 1.18 for sensitive) and Na^+^/K^+^ (11, 0.37, and 91.3 for tolerant vs. 31.4 and 47.6 for sensitive) and lower Phe (0.93 and 1.06 for tolerant vs. 1.45 and 2.0 for sensitive) and Pro ratios (0.71 and 0.75 for tolerant vs. 1.41 and 0.83 for sensitive). In the case of drought stress, tolerance correlates with increased ratios of Ile (1.87 and 1.1 for tolerant vs. 0.78 and 0.5 for sensitive), Gly (5.68 and 1.12 for tolerant vs. 0.85 and 0.5 for sensitive), Ser (2.72 and 1.17 for tolerant vs. 0.75 and 0.46 for sensitive) and Asn (2.12 and 1.38 for tolerant vs. 0.79 and 0.32 for sensitive), and decrease ratios of Hydric potential (1.63 and 1.33 for tolerant vs. 2.67 and 1.84 for sensitive) and Phe (1.26 and 1.03 for tolerant vs. 2.0 and 1.69 for sensitive) and Trp (1.08 and 1.79 for tolerant vs. 2.0 and 7.61 for sensitive) (Figure 7A, Supplementary Table 1). All the results are summarized in the form of a heat map in Figure 7B. The numerical data of the ratios of the Stress/control for all values are presented in Supplementary Table 1.

One interesting aspect of our results is that, among varieties, physiological parameters are not a distinctive trait for abiotic stress tolerance. Several previous studies have determined the effect of stress on melon physiology (Zhang et al., 2021). In a recent study on muskmelon genotypes under drought stress, the net photosynthetic rate, stomatal conductance (Gs), and the transpiration (E) rate decreased (Ansari et al., 2019). Other reports described similar decreases in stomatal conductance in genotypes different from the ones that we have used in this study (Kusvuran, 2012; Wang et al., 2016). There are reports indicating that during drought stress in melon, there was a significant increase in water use efficiency in drought tolerant genotypes (Akhoundnejad and Dasgan, 2019). In our case, under drought conditions, we did not observe any significant differences with the control values, that is, efficiency is maintained, although in this report plants were grown in field conditions and measures were taken in older plants.

We have also calculated the differential traits between Galia and Piel de Sapo irrespectively of their stress tolerance (Supplementary Figure 1). In this case, the Piel de Sapo variety showed higher Stress/control ratio for E, A, WUEintr and WUEinst under salt stress (Supplementary Figure 1). In agreement with our results, it has been previously described that under salt stress in muskmelon there is a significant increase in WUEintr and WUEinst with respect to control conditions (Ansari et al., 2018). Our data under saline stress conditions showed no changes for Galia plants, but we confirmed the increase in our conditions for Piel de Sapo cultivars. The fact that most of the differences observed in the physiological traits are variety dependent and not stress dependent may be explained by the differences in the leaf morphology.

There is no description available in the literature regarding the behavior of the free amino acid pools under salt and drought stress in *Cucumis melo* comparing stress and different varieties, so here we have investigated the complete free amino acid profile in our plants under the studied conditions. Salt tolerance correlated with higher levels of His in tolerant plants. His has been related to tolerance against heavy metals as it can chelate them, but its role in salt stress tolerance has not been described. Proline it is known to act as and osmolyte, but we have found that tolerant plants have less stress/control ratio than the sensitive ones, pointing out that the increases observed upon salt stress are not determinant for salt stress tolerance. In the case of drought stress, Ile, Gly, Ser and Asn were higher in tolerant varieties. Glycine can act as an osmolyte and also is a component of the tripeptide glutathione. Serine is also a precursor of cysteine and other stress-related molecules. On the other hand, high stress/control ratios of Phe correlated with sensitivity to stress, and it is the only molecule that decreased under conditions of both drought and salt stress. Phe is a precursor of several molecules, among them lignin, a pivotal molecule for cell wall biosynthesis. The accumulation of Phe in sensitive cultivars, irrespectively of the varieties, may be a symptom that basic plant processes like cell wall biosynthesis are more affected by stress than in tolerant cultivars.

Potassium is the major ion in the cytoplasm and thus is largely responsible for the intracellular ionic environment. Sodium is toxic for melon plants and must be extruded from the cell, or accumulated in the vacuole. Regarding ion accumulation, our results suggest that the limiting factor for stress response is the ability to accumulate sodium (Serrano et al., 1999; Rodríguez-Navarro, 2000). Under salt stress, plants can extrude sodium from the root, or take it up, transport it to the aerial part and accumulate it in the vacuoles (Arzani and Ashraf, 2016). The higher Na^+^ and Na^+^/K^+^ ratio of a salt-tolerant variety in our study may be explained by the vacuolar accumulation of sodium in the leaf tissues. Similar results regarding the Na^+^/K^+^ ratio and water use efficiency were observed in a field trial with *Cucumis melo cv. Huanghemi* (Tedeschi et al., 2017) so the trend is the same, even when we compare field/greenhouse conditions and early development/late develompment. Here we demonstrate that the ability to accumulate sodium and maintain a high Na^+^/K^+^ ratio is a distinctive trait for tolerant cultivars. We did not find any distinctive pattern with the potassium levels under drought stress. Therefore, the role of potassium as an osmolyte is not a limiting factor for drought tolerance in these melon cultivars.

Melon is a climacteric fruit, so its hormonal levels are subjected to drastic changes (Dunlap et al., 1996). There are several descriptions in the literature of the hormonal levels in cucurbit plants under abiotic stress. For instance, exogenous application of SA increases drought tolerance in muskmelon (Korkmaz et al., 2007), similar to what is observed in other cultivated plants (Souana et al., 2020). In addition, SA and JA levels increase upon spermidine addition and increase tolerance to salt stress (Radhakrishnan and Lee, 2013). Also, JA levels tend to increase in cucumber plants subjected to drought stress (Llanes et al., 2016). It has also been described that under mild or moderate water stress, IAA concentrations tend to increase (Huang et al., 2018). ABA is the main player in the abiotic stress response in plants, and it has also been described to increase upon abiotic stress in melon (Sun et al., 2013). When we studied the phytohormone levels, we did not find any common pattern among the sensitive or tolerant varieties and cultivars studied, although the concentrations of SA and IAA were higher in Piel de Sapo cultivars (between 3- and 7-fold for SA and 4- to 17-fold for IAA).

Taken together, we have performed a complete study of two different melon varieties comparing sensitive and tolerant cultivars and applied statistical tools to the results to find common patterns in salt or drought stress responses that could be useful to predict the behavior of uncharacterized varieties and cultivars and to design novel classical or biotechnological breeding strategies. Varieties or cultivars with increased His content and/or the ability to accumulate sodium (likely in the vacuoles) may display improved tolerance to salt stress, while novel varieties with enhanced levels of Ile, Gly, Ser, and Asn could show better performance under drought stress conditions. High levels of Phe seem correlate with diminished tolerance to abiotic stress. Thus, our results have provided a useful framework for future studies which will examine the ability of these parameters to predict stress tolerance performance in additional melon varieties and cultivars.

## Data Availability Statement

The raw data supporting the conclusions of this article will be made available by the authors, witho undue reservation.

## Author Contributions

SC, LD-E, and GM-S cultivated the plants, obtained the samples, and performed the experiments. LM and AV did all the physiological determinations and the calculations. JL-N did the amino acid measurements. LY and JM analyzed the results and designed the figures. JB did the statistical analysis and analyzed the results. GM-S, LY, and JM wrote the manuscript. JM designed the study. All authors contributed to the article and approved the submitted version.

## Funding

SC was a recipient of grant FPU19/01977 from the Spanish Ministerio de Universidades. LM and AV activities were funded by the Prometeu program (IMAGINA project, PROMETEU/2019/110). LM was also supported by the Spanish MICINN (PTA2019-018094). The CEAM foundation was funded by the Generalitat Valenciana.

## Conflict of Interest

The authors declare that the research was conducted in the absence of any commercial or financial relationships that could be construed as a potential conflict of interest.

## Publisher's Note

All claims expressed in this article are solely those of the authors and do not necessarily represent those of their affiliated organizations, or those of the publisher, the editors and the reviewers. Any product that may be evaluated in this article, or claim that may be made by its manufacturer, is not guaranteed or endorsed by the publisher.
