# Supplementary material for: Distinctive Traits for Drought and Salt Stress Tolerance in Melon (Cucumis melo L.)
Source: Front Plant Sci. 2021 Nov 4;12:777060. doi: 10.3389/fpls.2021.777060 (PMC8600367; doi:10.3389/fpls.2021.777060)

Supplemental FIGURE 1: Differences found in this study among different varieties. Radial diagrams of the ratio between stress/control concentration under salt stress (A) or stress/control concentration under drought stress (B). The Galia cultivars are shown in shades of blue and the *Piel de Sapo* cultivars in shades of red. The values are represented in a decimal logarithmic scale.


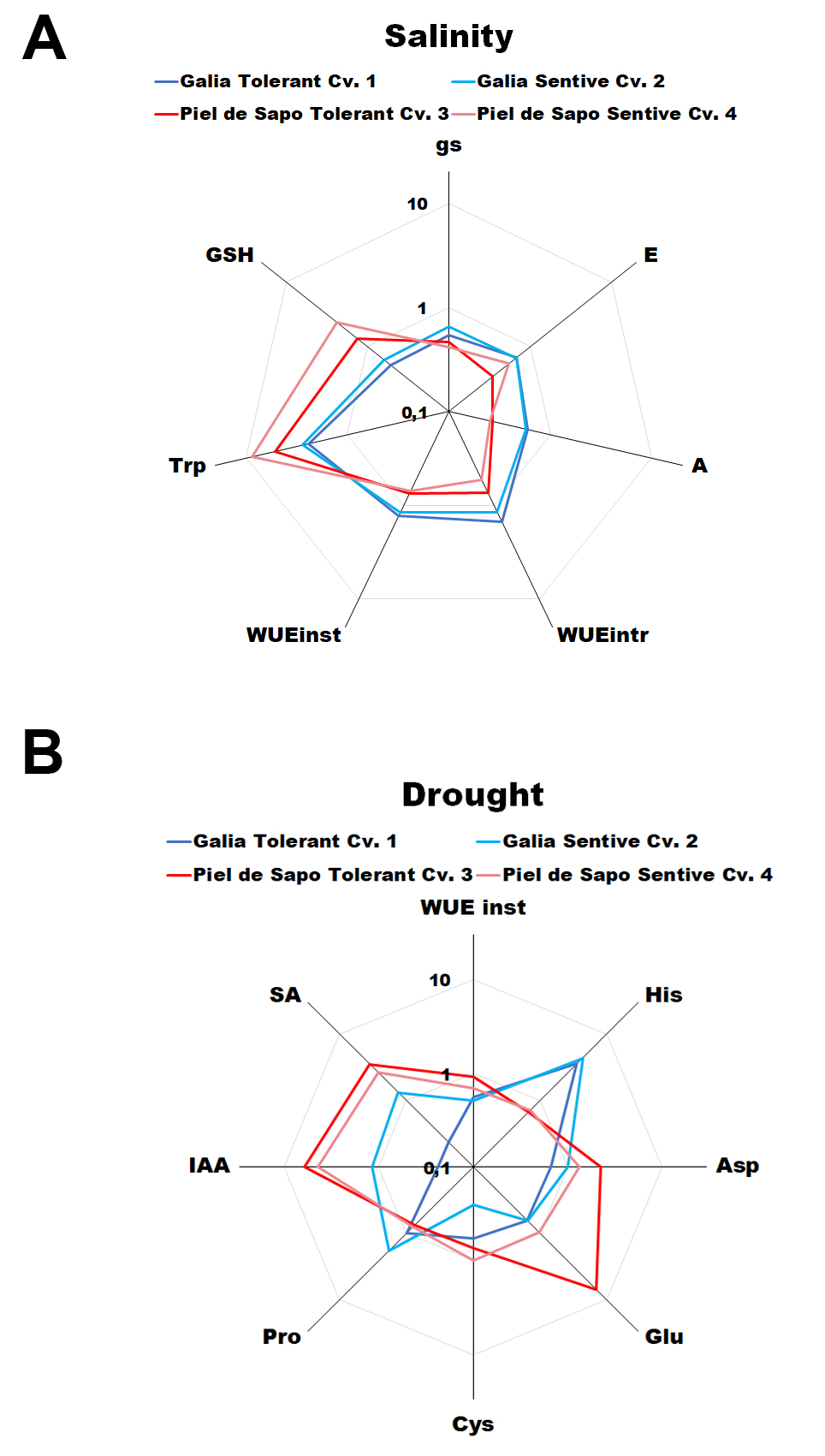

Supplement: Supplementary file 1 [file Table_1.DOCX]
