# Supplementary material for: Distinctive Traits for Drought and Salt Stress Tolerance in Melon (Cucumis melo L.)
Source: Front Plant Sci. 2021 Nov 4;12:777060. doi: 10.3389/fpls.2021.777060 (PMC8600367; doi:10.3389/fpls.2021.777060)

Supplemental table 1: Numerical values of the ratio between stress/control concentration under salt stress or stress/control concentration under drought stress for all the parameters evaluated in the current study.


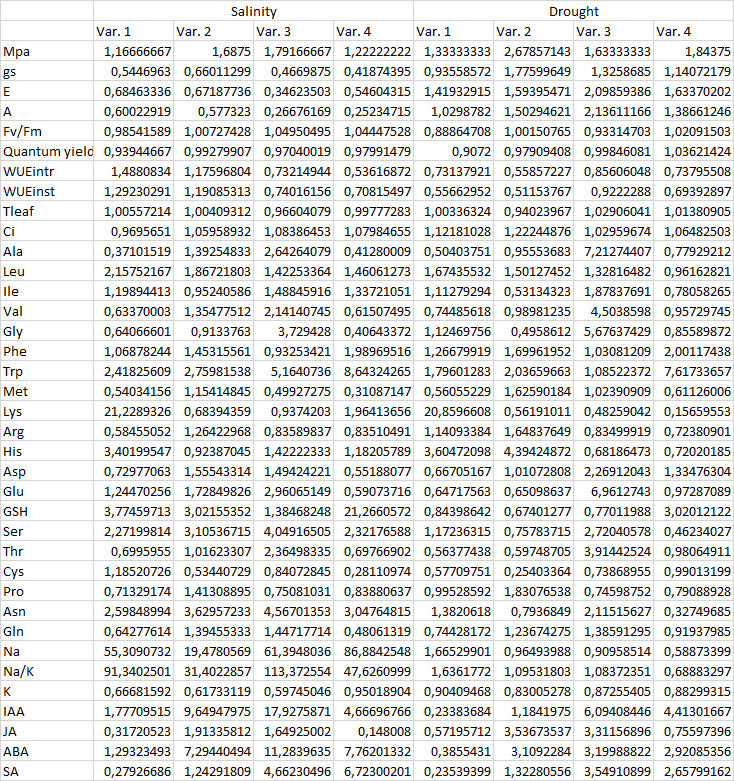

Supplement: Supplementary file 2 [file Table_2.DOCX]
